# Prevalence and associated factors of underweight among children 6–59 months of age in Takusa district, Northwest Ethiopia

**DOI:** 10.1186/s12939-018-0816-y

**Published:** 2018-07-24

**Authors:** Getnet Nigatu, Solomon Assefa Woreta, Temesgen Yihunie Akalu, Melaku Kindie Yenit

**Affiliations:** 1 Takusa District Health Office, University of Gondar, North-west, Gondar, Ethiopia; 2 Department of Health Informatics, Institute of Public Health, University of Gondar, Gondar, Ethiopia; 3 Department of Epidemiology and Biostatistics, Institute of Public Health, University of Gondar, Gondar, Ethiopia

**Keywords:** Underweight, 6–59 months children, Takusa, Northwest, Ethiopia

## Abstract

**Background:**

Most of the nearly 104 million underweight children in the world lived in South East Asia and sub-Saharan Africa in 2010. According to the 2014 Ethiopian Demographic and Health Survey (EDHS) report, 24 and 7% of children aged 6–59 months were underweight and severely underweight, respectively. Although appropriate child feeding and nutritional interventions reduce child illness and death, malnutrition remains a leading public health problem in Ethiopia. As literature on the issue is scarce in northwest Ethiopia, this study aimed at determining the prevalence of under-weight and associated factors in children 6–59 months of age in Takusa district, northwest Ethiopia.

**Methods:**

A community based cross-sectional study was conducted from January to February, 2017, at Takusa district, northwest Ethiopia. A total of 645 subjects were selected using the multi-stage sampling technique. Anthro software version 2.02 was used to determine the nutritional status of the children. A multivariable logistic regression analysis was used to investigate factors associated with underweight. Adjusted Odds Ratios (AOR) with the corresponding 95% Confidence Interval (CI) were used to show the strength of associations, and variables with *P*-values of < 0.05 were considered statistically significant.

**Results:**

In this study, the overall prevalence of underweight was 19.5% (95% CI: 16.4–22.8). According to the multivariate analysis, urban residence (AOR = 0.60; 95% CI: 0.38–0.95), no antenatal care (ANC) follow up (AOR = 1.59; 95% CI 1.01–2.52), and mothers age (over 35 years) (AOR = 0.62; 95% CI: 0.38–0.99) were significantly associated with lower odds of underweight.

**Conclusion:**

In the study community**,** the prevalence of underweight was lower than the findings of different studies in Ethiopia. Advanced maternal age (> 35 years), no antenatal follow up during pregnancy, and rural residence were significantly associated with underweight. Therefore, interventions targeting community management of acute malnutrition might be appropriate to manage the problem of underweight; efforts should also be intensified to reduce under-weight by focusing on identified determinants.

## Background

Nutrition adequate for the ages of infants and young children is essential for healthy growth, proper organ formation, and function, as well as for a strong immune system and neurological development [1]. Under-five children are the most susceptible age group for malnutrition, and nutritional status during childhood is a sensitive indicator of community health. Under-nutrition which can make children underweight, stunted, and wasted does not only increase the risk of infections, morbidity, and mortality but can also decrease mental and cognitive development. The effect of child malnutrition is long lasting and goes beyond childhood [1]. Underweight, defined based on weight for-age, is a composite measure of stunting and wasting and is recognized as the indicator for assessing changes in the magnitude of malnutrition over time. Wasting is the result of a recent failure to receive adequate nutrition and may be affected by recent episodes of infections, like diarrhea and other acute illnesses. Wasting indicates current or acute malnutrition, resulting from failure to gain weight or weight loss [2].

Underweight remains one of the most common causes of morbidity and mortality among children throughout the world [3, 4]. Globally, about 104 million children were under-weight in 2010, and the majority of these lived in sub-Saharan Africa and South Asia. Malnutrition is one of the leading causes of morbidity and mortality in children under the age of 5 years in developing countries. Every year, 3.5 million children die of malnutrition-related causes, of which underweight accounts for nearly 1 million [2, 5]. About two in five (38%) children in sub-Saharan Africa are underweight; 10.5% wasted, (2.2% severely), and 46.5% stunted (half of them severely) [4]. In Ethiopia, the prevalence of underweight was 24%, while in Amhara region, where the study was conducted, it was 28.4% [6]. Studies in Ethiopia report that under nutrition remains a public health problem and that its prevalence, in rural Ethiopia in general and Bure district in particular, has been 27 and 14.3%, respectively.

Though childhood malnutrition continues to be the leading public health problem in developing countries, it can occur as a result of a wide range of factors. Lack of dietary diversity and micronutrient-dense food consumption and poor child feeding practices contribute to the high rate of child under nutrition. Various reports also indicate that underweight in children is mainly caused by inadequate food intake [7, 8], repeated infections [9–11], low parental education [12–14], lack of sanitation [15], poor feeding practices [16], no ANC [17–19], residence [20], child rearing practices [21], economic [21, 22], social, and cultural factors [23].

In Ethiopia, malnutrition is a leading cause of child illness and death. Having been firmly committed to addressing food insecurity and under-nutrition, the Government established various multi-sectoral groups to coordinate and support efforts to step up rural economic development and food security [24–26]. Moreover, efforts to enhance good nutritional practices in rural areas expand health institutions, provide nutrition counseling and food focusing on appropriate child feeding and intervention have been made. However, underweight is still a problem and nearly one-third of the under-weight children are found in the Amhara Region, northwest Ethiopia, where literature is scarce [27]. Thus, this study aimed at determining the prevalence of under-weight and associated factors in Takusa district.

## Methods

### Study design and setting

A community-based cross-sectional study was conducted from January to February 2017 in Takusa district, northwest Ethiopia. The site is located at 738 km from Addis Ababa, the capital of Ethiopia and has an estimated population of 172,754, living in 25 kebeles (smallest administrative units). It was estimated that about 23,391 under-five children were found in the district. The data were collected from January to February 2017.

### Population, sample size, and sampling procedure

All mothers with children aged 6–59 months and lived in the selected kebeles for at least 6 months were included in the study. When households had more than one such child, one of them was selected using the lottery method. Out of the 25 kebeles in the district, five were randomly selected by the simple random sampling technique. For this particular study, the sample size was calculated using Epi-info version 3.7 by considering the following assumptions: the prevalence of underweight as 25.6%, 95% level of confidence, and 5% margin of error. Moreover a 10% non-response rate and a design effect of 2 were also used to yield the final sample size of 645.

### Data collection tool and procedures

A pretested, structured and interviewer administered questionnaire was used to collect data. Cronbatch’s alpha was used to check the consistency of the tool which turned out to be 87%. Six data collectors (clinical nurses) and two supervisors (health officers) were recruited for the task. To maintain consistency, the questionnaire was first translated from English to Amharic, the native language of the study area, and re-translated to English by professional translators and public health experts. A two-day intensive training was given to data collectors and supervisors on the objective of the study, confidentiality of information, how to take anthropometric measurements and techniques of conducting interviews. Data were collected at household levels and mother or care givers were the actual respondents. If the care giver or mother was not found, the data collector visited the household at least twice. The response rate was 100%. Initially, anthropometric measurements, like weight and height/length were measured, and age was taken from mothers/caretakers. Child length and height were measured according to child age. Child length was measured with the child lying down (in recumbent) position when they are under the age of 2 years. Thin clothes were used to cover the length board for child comfort. We used a length board (infantometer) and a height board (stadiometer) to measure length and height, respectively. Then, weights for age, weight for height, and height for age were determined using the software Anthro. Weight was measured using the Salter scale. Heavy shoes and clothes were removed during data collection. The height of children was also measured at Frankfurt position (touch occipital, shoulder, buttock, calf, and heel). Length measurement was also used for children less than 2 years of age.

For young children, unable to self-care especially, weight was checked together with their mothers or care givers. Then the mother weighed alone and the child’s weight was found by subtracting the mother’s weight from the total weight. In general, calibration of instruments and standardization techniques were used to avoid discrepancies. Before data collection, training was given on Salter scale with a capacity of 25 kg, and height or length. For children less than 2 years, length was used to check their anthropometric measurement. The tool was piloted on 5% of the total sample out of the study area.

### Measurements and study variables

Under weight, the outcome variable of this study, was measured using the anthropometric indicator of weight-for-age (WAZ) in the form of z-score, using the WHO Anthro 2006 software. The z-score depicted the deviation from the median weight of the child according to the World Health Organization (WHO) reference of the median of the growth standard curve. Under-weight was defined as weight-for-age (Z-score < − 2), using child growth standards published by WHO in 2006. Severe underweight was diagnosed if it was below − 3 SD. Variables such as age, sex, maternal and paternal educational status, occupation, family size plus maternal characteristics, like number of children ever born, ANC visits, birth order, health status during pregnancy, as well as morbidity status, like fever, diarrhea, measles and ARI were assessed.

### Data processing and analysis

Data were entered into Epi-info version 7 and exported to the Statistical Package for Social Sciences (SPSS) version 20 for analysis. Descriptive statistics, including frequencies and proportions were computed and presented using texts, graphs, and tables. Both bivariable and multivariate logistic regression models were carried out. Variables with a *p*-value of less than 0.2 in the bi-variable analysis were entered into the multivariable analysis. Both Crude Odds Ratio (COR) and Adjusted Odds Ratio (AOR) with 95% confidence intervals were estimated to show the strength of associations. The technique was a backward stepwise regression method. Finally, a *p*-value of less than 0.05 in the multivariable logistic regression analysis was used to identify variables significantly associated with underweight. For this study, the Hosmer and Lemeshow goodness of fit test which yielded a p-value greater than 0.05 was considered.

## Results

### Socio-demographic and economic characteristics

A total of 645 households were included in the study. Nearly half (46.2%) of the mothers were in the age range of 15–29 years. The mean age of mothers was 30.49 (SD 6.24) years. The majority, 629 (97.5%), of the households were Amhara by ethnicity. Three-hundred fifty- three (54.7%) of the mothers and 269 (41.7%) of the fathers were illiterate. Out of the total households, more than two-thirds, 453 (70.2%), lived in rural areas. About 336 (52.1%) of the children were male with a mean age of 21.85 months, and a standard deviation (SD) of 13.2. The majority, 85.9 and 92.6%, respectively) of the mothers were married Orthodox Christians. Almost three-quarters (74.3%) of the mothers were house wives. One-third, (32.6%) of the households had below ETB 500 monthly income. It was revealed that nearly half (47.9%) of the index children were female. About 16.6% of the mothers reported that their children suffered from diarrhea (Table 1).

**Table 1:** Socio-economic and demographic factors in Takusa district, North West Ethiopia, 2017

Variables	Frequency	Percent
Mother’s age (In years)		
15–29	298	46.2
30–34	165	25.6
≥ 35	182	28.2
Religion		
Orthodox	597	92.5
Others^a^	48	7.5
Marital status		
Not married	91	14.1
Currently married	554	85.9
Mother’s educational level		
Unable to read and write	353	54.7
Able to read and write	194	30.1
Primary and above	98	15.2
Husband’s educational level		
Unable to read and write	269	41.7
Able to read and write	251	38.9
Primary and above	125	19.4
Mother’s main occupation		
House wife	479	74.3
Government employee	62	9.6
Others^b^	104	16
Family monthly income (ETB)		
≤ 500	210	32.6
501–1000	233	36.1
≥ 1001	202	31.3
Family sizes		
≤ 4	304	47.1
5–7	273	42.3
8–11	68	10.5
Number of Under-five children		
≤ 2	500	77.5
> 3	145	22.5
Age of index child (In months)		
6-< 12	179	27.8
12-< 24	172	26.7
24-< 36	155	24.0
36-< 48	90	14.0
48–59	49	7.5
Sex of index child		
Male	336	52.1
Female	309	47.9
Household land ownership		
Yes	559	86.7
No	86	13.3
Child illness		
Diarrhea	107	16.6
Measles & ARI	39	6.0
No diseases	499	77.4

^a^prtotestant and Muslim, ^b^self employed, daily laborer

### Health service and environment related characteristics

The majority, 510 (79.1%), of the households had latrines. A large proportions, 266 (60.3%), of the rural households of the district used river water for drinking. About 504 (78.1%) of the households reported that they washed their hands after toilet; 160 (31.7%) of these were urban and 344 (68.3%) rural households. Three-fourths (76.3 and 77.7%, respectively), of the mothers had ANC follow-ups and took extra foods during pregnancy, and the majority (79.2%) had post-natal care visits after pregnancy (Table 2).

**Table 2:** Health service and environmental related factors in Takusa district, North West Ethiopia, 2017

Variables	Frequency	Percent
ANC follow-up		
Yes	494	76.6
No	153	23.4
ANC visits		
≤ 3 times	421	85.5
> 3Times	73	14.5
PNC visit		
Yes	514	79.7
No	131	20.3
Source of drinking water		
Piped water	441	69.8
Protected well and spring	139	22.1
Others (River and Unprotected well &Spring)	51	8.1
Distance to fetch drinking water		
Less than an hour	472	73.2
1 h or more	61	9.5
Water on premises	112	17.3
Have Latrine		
Yes	510	79.1
No	135	20.9
Hand washing with soap after toilet		
Yes	504	78.1
No	141	21.9

### Prevalence of underweight and feeding practices of children

In this study, the prevalence of underweight, stunting, and wasting were 126 (19.5%), 236 (36.5%), and 52 (8%), respectively. The proportion of severe and moderate underweight children was 53 (8.2%) and 73 (11.3%), respectively. Underweight was higher (76.9%) among rural dwellers than among urban residents (23.1%).

One-fifth, 133(20.6%), of mothers gave food and/or drink to the new-born before the establishment of breast milk in the first 3 days of delivery (prelactal feeds), but about 105 (16.3%) of the children did not get the first milk (the colostrum). The majority, 527 (81.7%), of the children were on exclusive breastfeeding, and about 58.8% started complementary feeding at 6 months. About 501 (77.7%) of mothers had extra food during pregnancy (Table 3).

**Table 3:** Nutritional status and Feeding practices of childrenin Takusa district, North West Ethiopia, 2017

Variables	Frequency	Percent
Underweight (WAZ)		
Normal (≥ -2SD)	519	80.5
Moderate (≥ − 3 < -2SD)	73	11.3
Severe (<-3SD)	53	8.2
Overall Underweight (<-2SD)	126	19.5
Stunting (HAZ)	236	36.6
Wasting (WHZ)	52	8.1
Complementary feeding started		
< 6 months	73	11.3
At 6 months	379	58.8
6–7 months	165	25.6
> 7 months	28	4.3
Frequency of complementary feeding per day		
< 3 times	96	14.9
3–4 times	407	63.1
> 4times	142	22.0
Duration of complementary feeding for child		
< 1 year	25	3.9
1–2 years	220	34.1
> 2 years	400	62.0
Types of food given for child		
Cereal based Porridge	72	11.1
Others (Cereal based Atmit, cow milk and formula milk)	81	12.6
Discard colostrum		
Yes	540	83.7
No	105	16.3
Time to start breast-fed		
Within 1 h	478	74.1
2–24 h	142	22.0
After a day	25	3.9
Exclusive breastfeeding		
Yes	527	81.7
No	118	18.3
Frequency of breastfeed per day		
< 4 times	43	6.7
4–7 times	231	35.8
8–10 times	268	41.6
> 10 times	103	16.0
Prelactal feeding		
Yes	133	20.6
No	512	79.4
Extra food during pregnancy		
Yes	501	77.7
No	144	22.3

### Factors associated with underweight among children aged 6–59 months

In the bivariate logistic regression analysis, marital status, mother’s occupation, ANC, age of mother, husband’s education, residence, comprehensive knowledge on IYCF, household income, breast-feeding during crying, child complementary feeding, frequency of breast feeding, and distance of water source were factors associated with underweight at a *p*-value of less than 0.2. Consequently, these variables were subjected to multivariate logistic regression analysis, and it was noted that residence, age of mother, and ANC follow up were significantly associated with underweight at a p-value of 0.05.

According to the multivariable logistic regression analysis, the odds of underweight children among urban dwellers were 39.6%, less likely to compare with those of rural residents (AOR = 0.604; 95% CI: (0.381–0.958). Similarly, the odds of underweight children among mothers who were above 35 years were 38.5%, unlike that of mothers below 35 years of age (AOR = 0.615; 95%CI: (0.382–0.99). Higher odds of underweight children were observed among mothers who had no antenatal care follow-up (ANC) (AOR = 1.595; 95% CI: (1.010–2.520) (Table 4).

**Table 4:** Factors associated with underweight among children aged 6–59 months in Takusa district, North West Ethiopia, 2017

Variables	Underweight		COR (95% CI)	AOR (95% CI)
	Yes	No		
Place of residence				
Rural	356(78.6%)	97(21.4%)	1	1
Urban	163(84.9%)	29(15.1%)	0.65(0.42–1.03)	0.60(0.38–0.95)^**a**^
Mother’s age				
15–34 years	366(79%)	97(21%)	1	1
> 35 years	153(84.1%)	29(15.9%)	0.78(0.48–1.28)	0.62(0.38–0.99)^**a**^
Mother’s occupation				
House wife	380(79.3%)	99(20.7%)	1	1
Others	139(83.7%)	27(16.3%)	0.89(0.52–1.52)	0.63(0.18–2.17)
Distance to fetch water				
< 1 h	372(78.8%)	100(21.2%)		1
1 h or more	51(83.6%)	10(16.4%)	0.73(0.36–1.48)	0.88(0.52–1.51)
Child Complement feeding				
< 6 month	205(77.1%)	61(22.9%)	1.43(0.97–2.13)	0.81(0.53–1.24)
> 6 month	314(82.8)	65(17.2%)	1	1
Comprehensive Knowledge on IYCF				
Yes	391(81.8%)	87(18.2%)	1	1
No	128(76.6%)	39(24.4%)	1.36(0.89–2.09)	1.44(0.92–2.27)
ANC follow up				
Yes	402(81.7%)	90(18.3%)	1	1
No	117(76.5%)	36(23.5%)	1.37(0.88–2.10)	1.59(1.01–2.52)^**a**^
Given breast milk during cry				
Yes	213(77.7%)	61(22.3%)	1.35(0.91–1.99)	1.92(0.83–1.92)
No	316(82.5%)	65(17.5%)	1	1

^a^Variable significant at *p*-value less than 0.05

## Discussion

In this study, the overall prevalence of underweight was 19.5% with a 95% Cl (16.4–22.8%). The finding was lower than those of Mecha district (34%) [28], Medebay Zana district (Tigray) (45.3%) [29], Haramaya (36.6, 28.2%) [30], in Ethiopia. This might be due to the fact that mothers in food insecure areas were at a high risk of getting underweight babies [7], while participants in this study lived in a food secure area that maximized the frequency of feeding, and making it possible for providing diversified food provision. Similarly, the finding was lower than findings overseas, for example, Bangladesh 43% [31], Yemen 46.2% [17], and Nepal 27.4% [32]. The possible justification for this could be variations among participants in wealth, access to health care, and differences in socio-demographic characteristics [17, 33]. For example, participants in Nepal came from hilly areas where low crop productivity minimized access to balanced nutrition and appropriate health care [32]. In contrast, the magnitude of underweight in our study was higher than 5.74% [34] and 14.9% [35] reported from Tanzania and Kenya, respectively. This difference might be due to the fact that participants from Tanzania and Kenya were urban dwellers and had access to more fruit, vegetables, and dairy products [8]. In addition, variations in study settings, high proportions of educated mothers [28, 32, 36], better wealth index [37], and access to health care [28] might be the other reasons for the variations.

Out of variables which showed significant associations with underweight lower odds of the problem were noted among urban dwellers. It was seen that the odds of underweight among urban children were 39.6%, highly unlikely to compare to rural ones. This finding was consistent with reports from Mozambique [36] and Bangladesh [38]. The notable difference in the rate of underweight among urban and rural children might be differences in living conditions, variations in early screening of mothers at child conception in urban areas compared with rural settings, exposure to poor dietary diversity [7, 8], and greater risks of infections among rural children [10]. Moreover, access to fruit and vegetables, and dairy products among urban dwellers might be the other factors for the differences in underweight rates [8]. In addition, most rural mothers spent their time in fields engaged farming activities and couldn’t offer a 24-h breastfeeding instead they provided only the usual food items which wouldn’t substitute the expected benefits [16, 22].

Like other studies conducted in Haramaya [30], Malawi [18], rural Yemen [17], and Somali Region [3], the odds of underweight in this study were high among mothers who had no antenatal care follow-ups. This might be due to the fact that accesses to health care services, like ANC, are important sources of information for women to access nutritional and health messages [18]. Furthermore, mothers who had ANC follow-ups had knowledge sharing opportunities for optimal infant and young child feeding (IYCF) [28]. As a result, children whose mothers had ANC were less likely to be underweight. In addition, mothers taking ANC were informed about breastfeeding which is of indispensable importance to minimize underweight among children [3]. Furthermore, variations in study settings and designs might be the other possible justifications for the observed differences.

The odds of underweight were low among children whose mothers were over 35 years when compared with those below. The finding was consistent with those of studies conducted in Belesa, and Yemen [39]. This could be due to the fact that young women are, by and large, less experienced in care giving and provide unqualified items [39]. Besides, adolescent mothers demand extra energy and nutrients for completing their growth and development. As a result, pregnancy during adolescence slows down the girl’s growth and may result in an underweight infant [40]. Therefore, this study has tremendous importance for both clinical and public health experts for further reducing burden of underweight. An assessment of a child’s nutritional status is an important routine indicator for monitoring growth and development. It helps to control death and complications relating to malnutrition. Moreover, from the public health perspective, reducing the prevalence of underweight is an important measure for providing supplementation activities to prevent further risks of underweight [2]. Though the study did its best to indicate the magnitude of underweight using a community-based investigation and a large sample, it was not free from limitations. For example, the cross-sectional design might have prevented the work from showing temporal relationships.

## Conclusion

In this community, the prevalence of under-weight was lower than the national figure. Advanced maternal age (> 35 years), antenatal care follow-up and urban residence were significantly associated with lower odds of the problem. Therefore, improving health service utilizations, such as ANC follow-up, access to information for rural areas are highly recommended.

## Abbreviations

ANC: Antenatal care
AOR: Adjusted Odds Ratio
CI: Confidence Interval
EDHS: Ethiopian Demographic Health Survey
EPI: Expanded Program on Immunization
MDG: Millennium Development Goal
SAM: Sever Acute Malnutrition
SUN: Scale Up Nutrition
UNICEF: United Nations Children Emergency Fund
